# Influence of Solute Size on Membrane Fouling during Polysaccharide Enrichment Using Dense Polymeric UF Membrane: Measurements and Mechanisms

**DOI:** 10.3390/membranes12020142

**Published:** 2022-01-24

**Authors:** Pooreum Kim, Hyungsoo Kim, Heekyong Oh, Joon-seok Kang, Sangyoup Lee, Kitae Park

**Affiliations:** 1 Graduate School of Water Resources, Sungkyunkwan University, Suwon 16419, Korea; poorerm@naver.com (P.K.); joonkeok0724@gmail.com (J.-s.K.); 2 School of Environmental Engineering, University of Seoul, Seoul 02504, Korea; phoh9676@gmail.com

**Keywords:** polysaccharide enrichment, membrane fouling, fouling mechanisms, Hermia model, dense UF membrane

## Abstract

Fouling mechanisms associated with membrane-based polysaccharide enrichment were determined using a dense ultrafiltration (UF) membrane. Dextran with different molecular weights (MWs) was used as a surrogate for polysaccharides. The influence of dextran MW on fouling mechanisms was quantified using the Hermia model. Flux data obtained with different dextran MWs and filtration cycles were plotted to quantify the more appropriate fouling mechanisms among complete pore blocking, standard pore blocking, intermediate pore blocking, and cake filtration. For 100,000 Da dextran, all four mechanisms contributed to the initial fouling. As the filtration progressed, the dominant fouling mechanism appeared to be cake filtration with a regression coefficient (R^2^) of approximately 0.9519. For 10,000 Da, the R^2^ value for cake filtration was about 0.8767 in the initial filtration. Then, the R^2^ value gradually decreased as the filtration progressed. For 6000 Da, the R^2^ values of the four mechanisms were very low in the initial filtration. However, as the filtration progressed, the R^2^ value for cake filtration reached 0.9057. These results clearly show that the fouling mechanism of dense UF membranes during polysaccharide enrichment can be quantified. In addition, it was confirmed that the dominant fouling mechanism can change with the size of the polysaccharide and the duration of filtration.

## 1. Introduction

Membrane processes are a major tool that have been widely used in food processing as well as water treatment fields, such as water treatment and wastewater treatment, over the past few decades [1]. Membrane technology has been applied to enrichment for processing fruit juices and beverages [2], and such enrichment has been reported to influence improvements in quality in related product applications [3]. Membrane-based enrichment technology is also becoming important in the membrane manufacturing industry and various water applications requiring high purity polysaccharides [4]. The consumption of high-quality food is increasing due to increased interest in human health and nutrition, and membrane processes have been reported as appropriate alternatives for enrichment processes [5]. In general, the membrane-based enrichment process for salt recovery uses nanofiltration (NF) and reverse osmosis (RO) or, more recently, forward osmosis (FO) membranes. However, UF is preferred for the enrichment of organic macromolecules such as polysaccharides and proteins by employing dense UF membranes (i.e., tight UF and loose NF membranes). Based on technological developments in ultrapure water pretreatment, the use of dense UF membranes is attracting attention in the food and pharmaceutical industry due to the ability to achieve the production of more treated water at lower operating costs. There is also a high interest in enrichment technology using dense UF membranes in the field of pathology, such as in the separation of plasma from blood, and in the field of micro-plastic removal, which is a recent major issue [6,7]. The membrane-based enrichment process has been shown to reduce the cost of electrical energy by applying low pressure, increase the product recovery rate, and reduce the cost of membrane cleaning and replacement due to membrane fouling [8]. In this respect, the use of dense UF membranes for the enrichment of useful macromolecules is becoming increasingly common.

Membrane technology could potentially be used globally in water treatment as well as in enrichment processes, but membrane fouling still remains a major hurdle. Therefore, in order to secure the efficiency of polysaccharide enrichment using a dense UF membrane, it is necessary to increase the understanding of membrane fouling. Some studies have shown that polysaccharides cause severe flux decline during membrane filtration processes [9]. Forms of membrane fouling vary and include adsorption, gelation, pore blocking, and cake layer formation [10]. However, until recently, little has been known of the fouling characteristics in dense UF membranes, particularly regarding membrane fouling caused by polysaccharides. In addition, research on the quantitative measurements of fouling mechanisms in this application is rather scarce [11,12].

In this study, fouling mechanisms associated with the membrane-based polysaccharide enrichment process were quantitatively determined using a dense polymer UF membrane (molecular weight cutoff (MWCO) of 6000 Dalton (Da)). The well-known Hermia model was used to determine the dominant fouling mechanisms, from among complete blocking, standard pore blocking, intermediate pore blocking, and cake filtration, by relating flux data to each fouling mechanism [13,14]. The dominant fouling mechanism for the size of polysaccharides (i.e., dextran) was quantified by comparing the correlation coefficient (R^2^) obtained by applying the Hermia model. In addition, the transformation of the fouling mechanism by the size of the polysaccharide and the duration of filtration was discussed.

## 2. Materials and Methods

### 2.1. Feed Water

Dextran (Sigma-Aldrich, Merck KGaA, Darmstadt, Germany) with different MWs of 6000, 10,000, and 100,000 Da was used as a surrogate for non-ionic polysaccharides. Table 1 lists the properties of the dextran used in fouling experiment. The concentration of dextran in feed water containing different sizes of dextran was adjusted to 1000 mg/L. The feed water pH was adjusted to 7.1 by adding hydrochloric acid or sodium hydroxide solution.

### 2.2. Membranes

The characteristics of the UF membrane used in this paper are listed in Table 2. The MWCO of the UF membrane is 6000 Da. The material and type of the membrane are polysulphone and hollow fiber, respectively (Asahi Kasei, Tokyo, Japan). The hollow fiber module was immersed in 1% sodium hypochlorite (NaOCl) solution and stored in a refrigerator before use. When examining the effect of dextran size on membrane fouling, virgin hollow fiber modules were used in membrane fouling experiments for each size of dextran.

### 2.3. Membrane Filtration System

**Figure 1:**
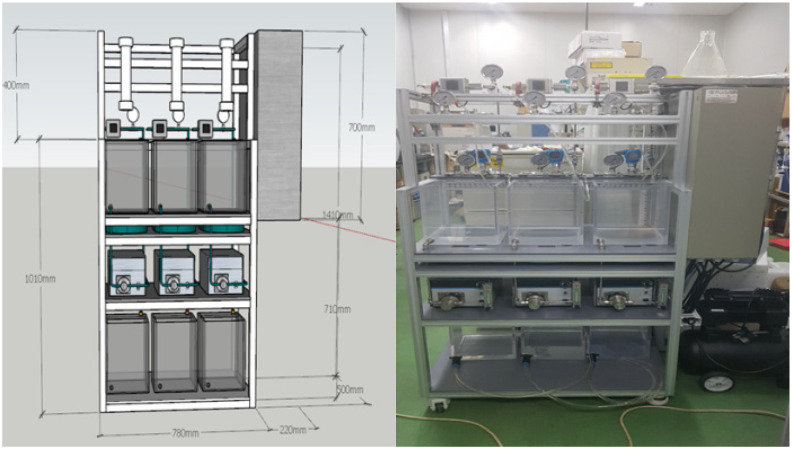
3D design and picture of membrane filtration system.

**Figure 2:**
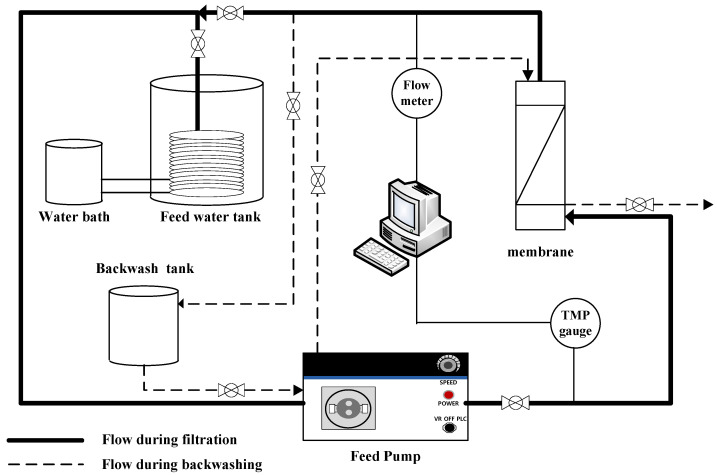
Schematic configuration of membrane filtration system.

A lab-scale filtration system employing the hollow fiber UF membrane module was used in this study. As shown in Figure 1, the filtration system consists of three parallel systems, allowing three different filtration experiments to be performed simultaneously and independently. As shown in Figure 2, each system consists of a 20-L feed water tank with a temperature controller, a metering pump (EMS0-2000S, Seoul, Korea), a stirrer, a digital flowmeter, a digital pressure gauge, and several valves for flow direction control (e.g., filtration and backwash). Both permeate flow and trans-membrane pressure (TMP) can be monitored and recorded continuously and automatically during filtration experiments using a data logging system installed on a laptop connected to the filtration system.

### 2.4. Operating Conditions

The TMP of each filtration was set to 1 bar and the flux decline due to fouling was monitored and recorded. The feed water dextran concentration was adjusted to 1000 mg/L. To minimize the effect of temperature changes, a stainless steel coil connected to the water bath was immersed in the feed water tank and the feed water temperature was kept constant at 20 ± 0.5 °C. The fouling experiment was conducted so that one cycle of filtration-backwashing was repeated four times (i.e., four cycles). At the end of each cycle, backwashing was performed by injecting backwashing water in the opposite direction of normal filtration through a feed pump. Backwashing was performed for 30 s by applying twice the normal filtration flow rate. Before starting a new cycle, the membrane was flushed using deionized (DI) water.

### 2.5. Measurement Fouling Mechanisms

The resistance-in-series model is frequently used to determine the fouling mechanism [15,16]. Solvent permeate flux (*J*) in cake filtration theory can be represented by Darcy’s law. Therefore, the degree of membrane fouling can be estimated by calculating the total resistance (*R_t_* = *R_m_* + *R_a_* + *R_p_* + *R_g_* + *R_c_*) using Equation (1), where ΔP is the TMP, μ is the dynamic viscosity, *R_m_* is the membrane resistance, *R_a_* is the resistance by adsorption, *R_p_* is the resistance by intramembrane plugging, *R_g_* is the resistance by gelation, and *R_c_* is the resistance by cake layer formation.

(1) $$J=\Delta P/\left(\mu \left(R_{m}+R_{a}+R_{p}+R_{g}+R_{c}\right)\right)$$

Given that most fouling is formed in a cake layer (*R_a_* = *R_p_* = *R_g_* = 0), Equation (1) can therefore be simplified as Equation (2):

(2) $$J=\Delta P/\mu \left(R_{m}+R_{c}\right)\;$$

Assuming that *R_c_* is formed at the same pressure, when comparing the mass of foulants accumulated on the membrane surface, *R_c_* can be determined by Equation (3) [17,18,19], where α is the specific cake resistance, *C_b_* is the mass of dry cake solids per volume of filtrate, *V* is the cumulative permeate quantity, and *A* is the effective membrane surface area.

(3) $$R_{c}=\left(\alpha C_{b}V\right)/A$$

The Hermia model has been reported to be an appropriate model for using permeate flux to explain the fouling mechanism in membrane filtration [20]. In this study, the membrane fouling mechanism was analyzed by quantifying the filtration resistance for each filtration cycle by applying the filtration resistance to the Hermia model after repeating a certain filtration cycle at a fixed TMP. The analysis of fouling mechanisms was performed using the following four models: the complete pore blocking model, the intermediate pore blocking model, the standard pore blocking model, and the cake filtration model.

The complete pore blocking model can be expressed by Equation (4), where *J* is the permeate at time *t* flux, *J*_0_ is the initial permeate flux, and *K_b_* is the characteristic fouling parameter. The complete pore blocking mechanism occurs when the solute size is greater than that of the membrane pores. As a result, solute particles do not enter into the membrane pores and do not permeate through the membrane.

(4) $$\ln J=\ln J_{0}-K_{b}t$$

The intermediate pore blocking model can be expressed by Equation (5), where *K_i_* is the characteristic fouling parameter. The intermediate pore blocking mechanism occurs when the solute size is similar to that of membrane pores, leading to the membrane pores being blocked near their entrance on the feed side. Additionally, the probability that the solute particles will block the membrane pores continuously decreases.

(5) $$1/J=1/J_{0}-K_{i}t$$

The standard pore blocking model can be expressed by Equation (6), where *K_s_* is the characteristic fouling parameter. The standard pore blocking mechanism occurs when the solute size is smaller than that of the membrane pores, which leads to internal pore blocking.

(6) $$1/J^{1/2}=1/J_{0}^{1/2}-K_{s}t$$

The cake filtration model can be expressed by Equation (7), where *K_c_* is the characteristic fouling parameter. The cake formation mechanism occurs when the solute size is much greater than the pore size; hence they are unable to enter the membrane pores. Factors affecting this type of mechanism are solute deformation, cake compression, and cake/gel layer thickness.

(7) $$1/J^{2}=1/J_{0}^{2}-K_{c}t$$

Moreover, the reversibility and irreversibility of membrane fouling can be analyzed using Equations (8)–(10). In these equations, the filtration resistance obtained by filtering DI water through the virgin membrane, the filtration resistance of the fouled membrane obtained after the filtration using the treated feed water, and the filtration resistance obtained in the re-filtered sample using DI after the physical cleaning were defined as *R*_0_, *R*_1_, and *R*_2_, respectively, where *RF* refers to reversible fouling, *IF* refers to irreversible fouling, and *TF* refers to total fouling.

(8) $$RF=\left(R_{2}-R_{1}\right)/\left(R_{0}-R_{1}\right)$$

(9) $$IF=\left(R_{0}-R_{2}\right)/(R_{0}-R_{1})$$

(10) TF=RF+IF=1

## 3. Results and Discussion

### 3.1. Fouling Tendency with Respect to Dextran Size and Filtration Cycle

**Figure 3:**
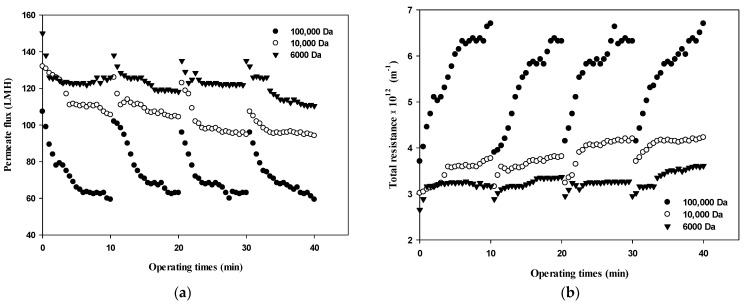
Changes in (**a**) permeate flux and (**b**) total resistance with respect to the dextran MW and filtration cycle.

Changes in the permeate flux and the filtration resistance with respect to the size of dextran and the filtration cycle (i.e., four cycles with one cycle of filtration and backwashing) are shown in Figure 3. In terms of operating conditions, the filtration experiment was performed at a constant pressure of 1 bar according to the standard certificate provided by the manufacturer. Various studies have shown that the degree of membrane fouling varies depending on the particle size, and it is very important to maintain constant flux and to introduce sustainable appropriate flux due to economic considerations [21]. As shown in Figure 3a, during the first cycle (i.e., initial 10 min), the flux decreased from 100 LMH to 60 LMH for 100,000 Da, from 130 LMH to near 100 LMH for 10,000 Da, and from 140 LMH to 120 LMH for 6000 Da. Based on the flux data obtained in Figure 3a, the total filtration resistance was calculated using Equation (1) and the results are illustrated in Figure 3b. Figure 3 clearly shows that flux decline due to dextran fouling is most severe for 100,000 Da dextran, with the highest fouling resistance. 

In addition, as the filtration cycle progressed, it was found that membrane fouling was more severe in the small dextran than in the large dextran. This implies that fouling mechanisms differ with respect to the size of dextran as well as the filtration duration. It can be seen that the difference in the degree of flux decline and filtration resistance according to the size of dextran and filtration cycle is related to the thickness, compactness, and compressibility of the fouling layer. It has been reported that the degree of fouling varies depending on the particle size and that microparticles find it difficult to form high resistant fouling layers [22]. Therefore, it is necessary to determine the dominant fouling mechanisms considering the size of foulants as well as the reversibility of fouling in terms of hydraulic backwashing.

### 3.2. Comparision of the Degress of Fouling Using Flux Nomalization for Different Sizes of Dextran

**Figure 4:**
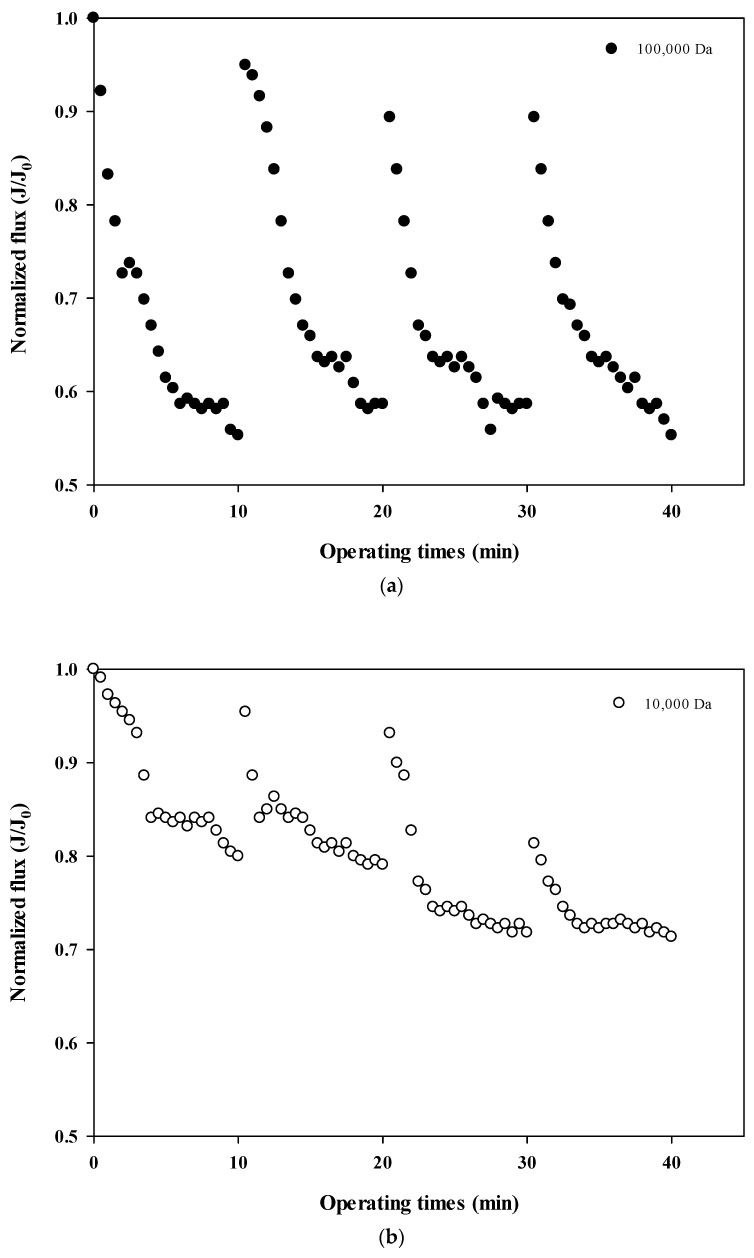

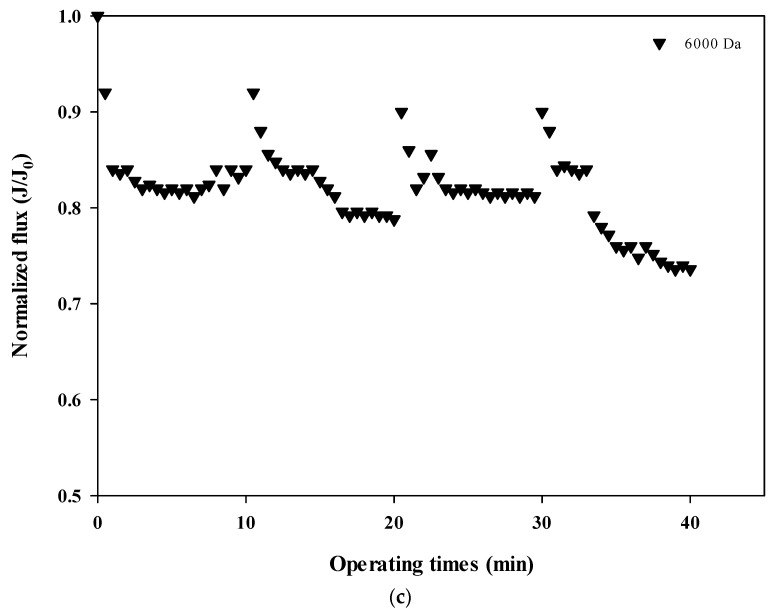
(**a**). Normalized flux with respect to filtration cycle for 100,000 Da dextran. (**b**). Normalized flux with respect to filtration cycle for 10,000 Da dextran. (**c**). Normalized flux with respect to filtration cycle for 6000 Da dextran.

The normalized fluxes are shown in Figure 4a (100,000 Da dextran), 4b (10,000 Da dextran), and 4c (6000 Da dextran), which can be used to compare the size-dependent fouling mechanism of dextran and the change of the fouling mechanism according to the progress of fouling. Figure 4a shows that the flux decreases the most at 100,000 Da, and Figure 4b,c show that the flux decreases relatively more as the cycle progresses. These results indicate that the flux does not tend to return to its initial flux as the filtration cycle progresses. This means that the degree of flux recovery by backwashing decreased and irreversible fouling increased with the progress of the filtration cycles. In addition, it was shown that the flux decreased every filtration cycle regardless of the dextran size, which means that fouling occurred mostly on the membrane surface rather than on the inside of the membrane pores. 

In addition, the degree of fouling reversibility according to hydraulic backwashing for each filtration cycle differs with the size of dextran. It seems that fouling is severe for the larger particles (i.e., 100,000 Da dextran) but irreversible fouling tends to occur with smaller particles (i.e., every cycle for 10,000 Da dextran and fourth cycle for 6000 Da dextran). These results suggest that the degree of fouling and the mechanisms involved in membrane fouling vary with respect to the size of the particle and the filtration duration. It has been reported that there is less tendency for fouling increases in microparticles and that fouling is only slightly affected by the size of microparticles [22,23]. It should be noted that in that study, hydraulic backwashing instead of chemical enhanced backwashing (CEB) was carried out between each cycle. This implies that fouling reversibility with respect to particle size could be different from the results obtained in this study when applying CEB.

### 3.3. Analysis Fouling Mechanisms for Different Sizes of Dextran Using Hermia Model

**Figure 5:**
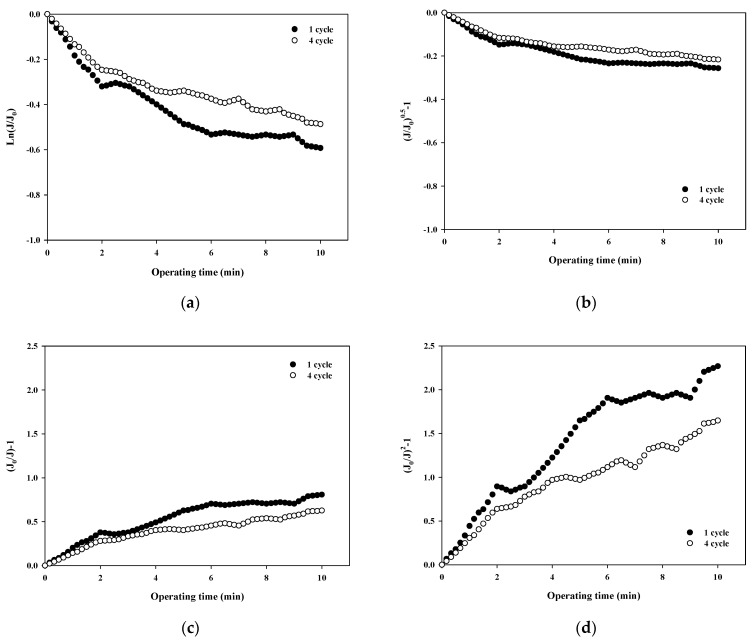
Analysis of fouling mechanisms for 100,000 Da dextran: (**a**) complete blocking, (**b**) standard pore blocking, (**c**) intermediate pore blocking, and (**d**) cake filtration.

The normalized flux data (i.e., first and fourth cycles) shown in Figure 4a for 100,000 Da dextran were used to quantify the fouling mechanism using the Hermia model (Equations (4)–(7)). The results shown in Figure 5a–d correspond to the complete pore blocking, standard pore blocking, intermediate pore blocking, and cake filtration models, respectively. The correlation coefficients, R^2^, for each model are listed in Table 3 for the initial and final cycles. By comparing the R^2^ values for each fouling model, the main fouling mechanism can be quantified (i.e., the higher the R^2^, the more significant the fouling mechanism). In Figure 5a, for the complete pore blocking model, the R^2^ showed a high correlation with 0.8659 in the first cycle, and the R^2^ slightly increased to 0.8895 in the fourth cycle. In Figure 5b, for the standard pore blocking model, the R^2^ was 0.8438 for the first cycle and 0.8686 for the fourth cycle. In Figure 5c, for the intermediate pore blocking model, the R^2^ was 0.9024 and 0.9252 for the first and fourth cycles, respectively. Lastly, in Figure 5d, for the cake filtration model, the R^2^ was 0.9284 for the first cycle and increased to 0.9519 for the fourth cycle. The results show that all four fouling mechanisms contribute to some extent to the flux decline and the filtration resistance increase during fouling with relatively higher R^2^ values above 0.84. In addition, the dominant fouling mechanism appears to be cake filtration as fouling progresses. This is in good agreement with the theory of the Hermia model that cake filtration occurs when the particle size is larger than the pore size of the membrane. However, it should be noted that the compressibility of the cake layer is rarely taken into account in this model. It is shown in Figure 5 that the degree of fouling changes with the filtration cycle with higher R^2^ values for the fourth cycle. This might be a result of the more compressible cake layer for the large dextran.

**Figure 6:**
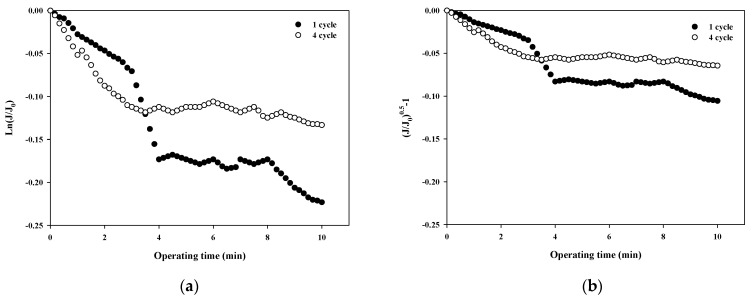

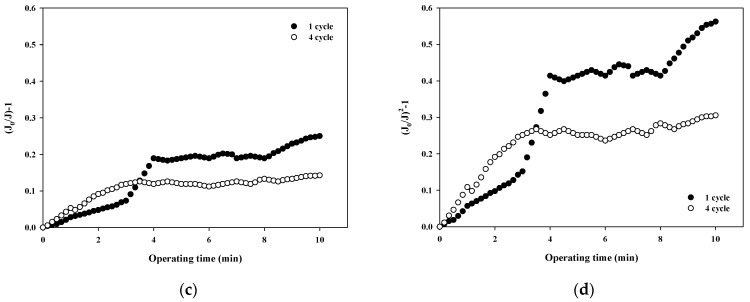
Analysis of fouling mechanisms for 10,000 Da dextran: (**a**) complete blocking, (**b**) standard pore blocking, (**c**) intermediate pore blocking, and (**d**) cake filtration.

The fouling mechanism was quantified using the normalized flux data (i.e., first and fourth cycles) shown in Figure 4b for 10,000 Da dextran. The results are shown in Figure 6. Interestingly, in all cases, the R^2^ was higher than 0.85 in the first cycle, with the highest R^2^ value for the cake filtration mechanism. However, the R^2^ value for all correlations decreased to less than 0.66. This means that the fouling mechanism changes with filtration time due to changes in the thickness, compactness, and compressibility of the fouling layer.

**Figure 7:**
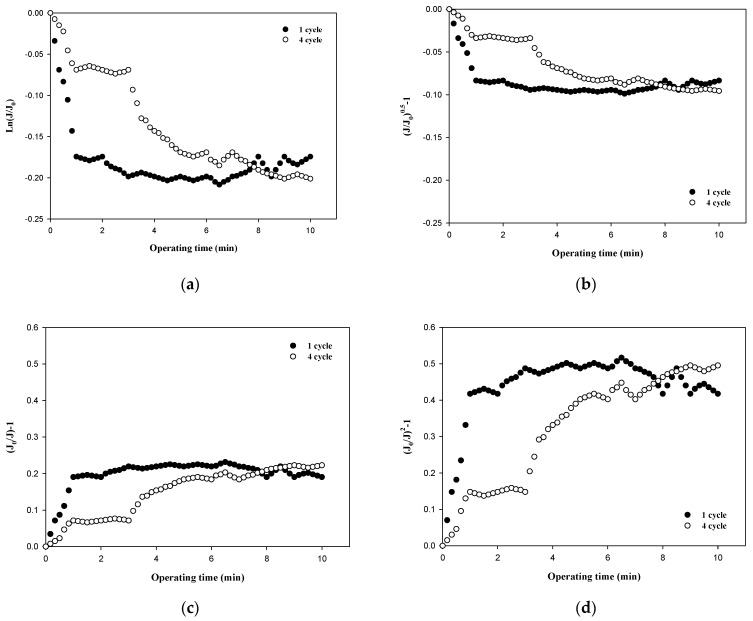
Analysis of fouling mechanisms for 6000 Da dextran: (**a**) complete blocking, (**b**) standard pore blocking, (**c**) intermediate pore blocking, and (**d**) cake filtration.

The fouling mechanism was quantified using the normalized flux data (i.e., first and fourth cycles) shown in Figure 4c for 6000 Da dextran. The results are shown in Figure 7. All four fouling mechanisms showed R^2^ values of less than 0.23 in the first filtration cycle, even though this small dextran caused noticeable flux decline, as shown in Figure 3. However, the R^2^ values increased significantly for the fourth cycles, to above 0.88. This also implies that the fouling mechanisms change as fouling progresses. With the exception of the largest dextran with a MW of 100,000 Da, it is difficult to specify a dominant fouling mechanism, and all four mechanisms appear to contribute to fouling to some extent.

**Figure 8:**
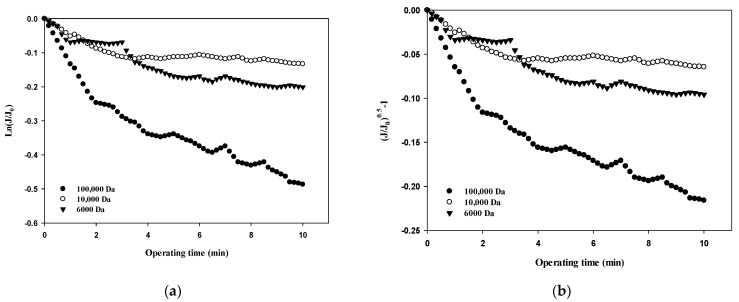

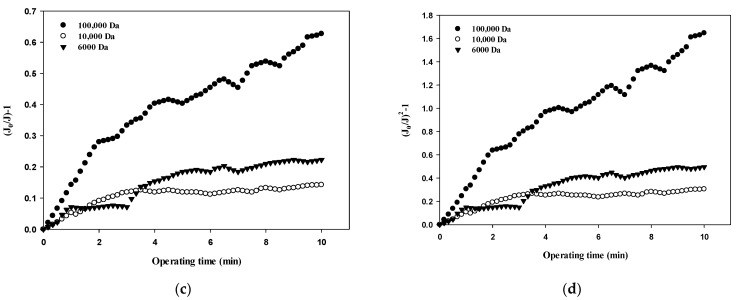
Comparison of fouling mechanisms with respect to the size of dextran: (**a**) complete blocking, (**b**) standard pore blocking, (**c**) intermediate pore blocking, and (**d**) cake filtration.

Table 3 summarizes the R^2^ values obtained from the results shown in Figure 5, Figure 6, Figure 7 and Figure 8 and compares the correlation results with respect to the fouling mechanisms for different sizes of dextran. The results for 100,000 Da of dextran show that all models are incompatible in the first cycle during early filtration, but that the effects of standard pore blocking drastically decrease for the fourth cycle. In addition, cake filtration becomes a major fouling mechanism. It has been reported that in the early stage of membrane fouling, particle fouling generally represents pore blocking and the level of blocking decreases with filtration time [24]. The results from this study show that pore blocking was highly correlated in the first cycle but that cake filtration showed a considerably high correlation for the fourth cycle. Regarding the results for 10,000 Da of dextran, the cake filtration model showed a relatively high correlation in the first cycle. However, cake filtration became less correlated for the fourth cycle. The results for 6000 Da of dextran showed that all four models were not compatible in the early filtration stage. Although correlations were not found in the early filtration stage, the correlation substantially increased for the fourth cycle, and the highest correlation was found with the cake filtration. It has been reported that the use of polysaccharides in membrane filtration processes could cause severe fouling due to the conformational changes in fouling layer, and that the fouling mechanism varies depending on the size of polysaccharides [25]. As reported previously, the dominant mechanism of polysaccharide fouling is cake layer formation [26]. Polysaccharide fouling is also strongly influenced by the transmembrane pressure, which affects the compressibility of the fouling layer, especially for large MWs [27]. The results obtained in this study are qualitatively in accordance with these previous studies, and suggest a way of quantitatively measuring fouling mechanisms with respect to the size of particles and the filtration time.

## 4. Conclusions

In this study, fouling mechanisms associated with the membrane-based polysaccharide enrichment process were quantitatively determined using dense polymeric ultrafiltration (UF) membranes. Flux data obtained during dextran fouling were correlated with the Hermia models, including for complete pore blocking, standard pore blocking, intermediate pore blocking, and cake filtration. The influence of the size of dextran and the filtration cycle on the fouling mechanisms were elucidated. The main results derived from this study are as follows. 

It was shown that the most significant flux decline occurred for 100,000 Da dextran, and the least amount of flux recovery after backwashing was obtained with 6000 Da dextran. Therefore, it is necessary to determine the dominant fouling mechanisms considering the size of foulants as well as the reversibility of fouling in terms of hydraulic backwashing. For dextran with a MW of 100,000 Da, all four mechanisms appear to have contributed to the initial fouling. As the cycle progressed, the dominant fouling mechanism appeared to be cake filtration with a regression coefficient (R^2^) of approximately 0.9519. For dextran with a MW of 10,000 Da, the R^2^ value for cake filtration was about 0.8767 in the initial filtration cycle. Then, the R^2^ value gradually decreased as the filtration cycle progressed. For dextran with a MW of 6000 Da, the R^2^ values of the four mechanisms were very low in the initial filtration cycle. However, as the cycle progressed, the R^2^ value for cake filtration reached 0.9057, indicating that cake filtration is the dominant fouling mechanism. These results imply that the degree of fouling depends not only on the size of particles but also the thickness, compactness, and compressibility of the fouling layer.

In addition, the dominant fouling mechanisms during fouling could be changed with filtration time, as there might be a conformational change in fouling layer. The results obtained in this study are qualitatively in accordance with the previous studies, and suggest a way of qualitatively measuring fouling mechanisms with respect to the size of particles and filtration time [28,29]. Through the results of this study, the membrane fouling mechanisms that occurs when a dense UF membrane is used in relation to polysaccharide enrichment was quantitatively determined. Overall, this study suggests that the fouling mechanism during polysaccharide enrichment using dense UF membranes can be quantified using the Hermia model and that the size of polysaccharides plays an important role in membrane fouling. A possible follow-up study could be to elucidate the membrane fouling mechanism when polysaccharides of various sizes are present in admixture and when ionic polysaccharides (i.e., alginate) are present.

## Figures and Tables

**Table 1:** Property of dextran.

Parameters	Values
Product name	Dextran
Molecular weight (Da)	6000; 10,000; 100,000
Appearance (Color)	White
Appearance (Form)	Powder
Loss on drying (%)	≤10

**Table 2:** Specifications of UF membrane.

Parameters	Values
Hollow fiber membrane size (mm)	0.6 (ID)
Effective membrane area (m^2^)	34
Molecular weight cutoff (Da)	6000
Membrane type	Hollow fiber
Membrane material	Polysulphone
pH range	1–14

**Table 3:** R^2^ values of Hermia’s models.

	Complete Blocking	Standard Pore Blocking	Intermediate Pore Blocking	Cake Filtration
100,000 Da (first cycle)	0.8659	0.8438	0.9024	0.9284
100,000 Da (fourth cycle)	0.8895	0.8686	0.9252	0.9519
10,000 Da (first cycle)	0.8624	0.85885	0.8698	0.8767
10,000 Da (fourth cycle)	0.6397	0.6349	0.6493	0.6587
6000 Da (first cycle)	0.2229	0.2224	0.2234	0.2234
6000 Da (fourth cycle)	0.8878	0.8828	0.8971	0.9057

## Data Availability

The data presented in this study are available on request from the corresponding author.
